# Dataset of ultrasound-assisted extraction of anthocyanin from the petals of *Clitoria ternatea* using Taguchi method and effect of storage conditions on the anthocyanin stability

**DOI:** 10.1016/j.dib.2022.107803

**Published:** 2022-01-06

**Authors:** Meng Hong Shu, Kanagesvari K. Annamalai, Farhana Nazira Idris, Azlina Harun Kamaruddin, Masrina Mohd Nadzir

**Affiliations:** School of Chemical Engineering, Engineering Campus, Universiti Sains Malaysia, 14300 Nibong Tebal, Pulau Pinang, Malaysia

**Keywords:** Anthocyanins, *Clitoria ternatea*, Green extraction, Green solvent, Storage stability, Taguchi method, Ultrasound-assisted extraction

## Abstract

Anthocyanins are natural water-soluble colourants with a number of reported health benefits and are an excellent alternative to artificial dyes. Anthocyanins from *Clitoria ternatea* were obtained using ultrasound-assisted extraction with glycerol-water (60:40 w/v). The anthocyanins in the extract were measured at wavelength 550 and 700 nm using a UV-visible spectrophotometer and expressed in terms of total anthocyanins content (TAC). Taguchi method was employed to optimize the extraction parameters that include the extraction time (30 to 50 min), extraction temperature (40 to 60 °C), and solvent to solid ratio (10:1 to 30:1), with TAC as the response. The obtained data showed the optimum extraction parameters as 30 min extraction time, 50 °C extraction temperature, and 10:1 solvent to solid ratio. The anthocyanin's storage stability was analyzed for 7 days at varying storage temperatures and exposure to light. The present dataset in this article indicated the glycerol-water system as a green alternative for anthocyanin extraction and acted as a storage medium. Furthermore, our methodology can be applied to optimize the anthocyanins extraction process, manipulate storage conditions and improve the extract quality.

## Specifications Table

**Table:** 

Subject	Food technology
Specific subject area	Anthocyanin extraction
Type of data	Table Figure
How data were acquired	Ultrasound-assisted extraction (UAE) using ultrasonic bath (Elmasonic, S80H) UV-visible spectrophotometer (Agilent Technologies, Cary 60) Total anthocyanins content (pH differential method) Taguchi method (Minitab, State College, PA, USA) Analysis of variance (ANOVA) using Minitab 18 statistical software (State College, PA, USA)
Data format	Raw Analyzed
Parameters for data collection	Water and glycerol-water (60:40 w/v and 90:10 w/v ) were used for the solvent screening process. Parameters for solvent screening in UAE were 40 °C extraction temperature, 15 min extraction time, and 10:1 solvent to solid ratio. Parameters for the optimization of UAE using Taguchi method were extraction time (30, 40, and 50 min), extraction temperature (40, 50, and 60 °C), and solvent to solid ratio (10:1, 20:1, and 30:1), and the response was TAC. TAC was expressed in terms of the differences in the absorbance (wavelength 550 and 700 nm) between pH 1.0 and 4.5. Parameters for the storage stability were temperature (4 and 25 °C) and lighting conditions (fluorescent light and dark). The percentage of anthocyanins degradation was expressed in terms of absorbance differences at wavelength 550 nm during 0, 1, 3, 6, and 7 days of storage.
Description of data collection	UAE with glycerol-water (60:40 w/v) was used to prepare the anthocyanins extract from the dried powder of *C. ternatea* flowers. Taguchi method was used to optimize the extraction condition of UAE. The absorbance value of anthocyanins in the extract was measured at wavelength 550 nm. TAC was quantified using the spectrophotometric pH differential method. Storage stability of anthocyanins extract was performed at different temperature and lighting condition. All samples were stored in a tightly sealed glass bottle and fully covered with aluminum foil except for fluorescent light condition. Absorbance readings at wavelength 550 nm were taken at 0, 1, 3, 6, and 7 days of storage for the percentage of anthocyanins degradation analysis. The significant difference of data and experimental error were determined by ANOVA using Minitab 18 statistical software. All experiments were conducted in triplicates (*n* = 3).
Data source location	Collection of fresh *C. ternatea* flowers: Private residence in Baling, Kedah, Malaysia Extraction and data analysis: School of Chemical Engineering Universiti Sains Malaysia, Engineering Campus 14300 Nibong Tebal, Pulau Pinang, Malaysia 5°08′47.2″N 100°29′31.0″E 5.146450, 100.491930
Data accessibility	Raw and processed dataset have been uploaded in Mendeley Data with DOI: 10.17632/xp8jvh3t2x.1 at this link: https://data.mendeley.com/datasets/xp8jvh3t2x/1

## Value of the Data

• The dataset of anthocyanins obtained from UAE from the *C. ternatea* flowers with glycerol-water system can be used as a reference to enhance the extraction efficiency of bioactive compounds in terms of quantity and quality compared to other types of extraction method.
• The data provided in this article can be used by natural food colourant producers and the food industry to optimize the anthocyanins extraction process and storage conditions.
• The data showed the extractability and storage ability of bioactive compound using glycerol-water system and the potential application of this solvent in the food, cosmetic, nutraceutical and pharmaceutical industry for developing new natural products.
• The dataset of TAC and the absorbance reading of anthocyanins extract from *C. ternatea* can serve as a benchmark for the analysis of anthocyanins-related compounds from different types of plant, fruit, berries, herb and spices.

## 1 Data Description

**Fig. 1:**
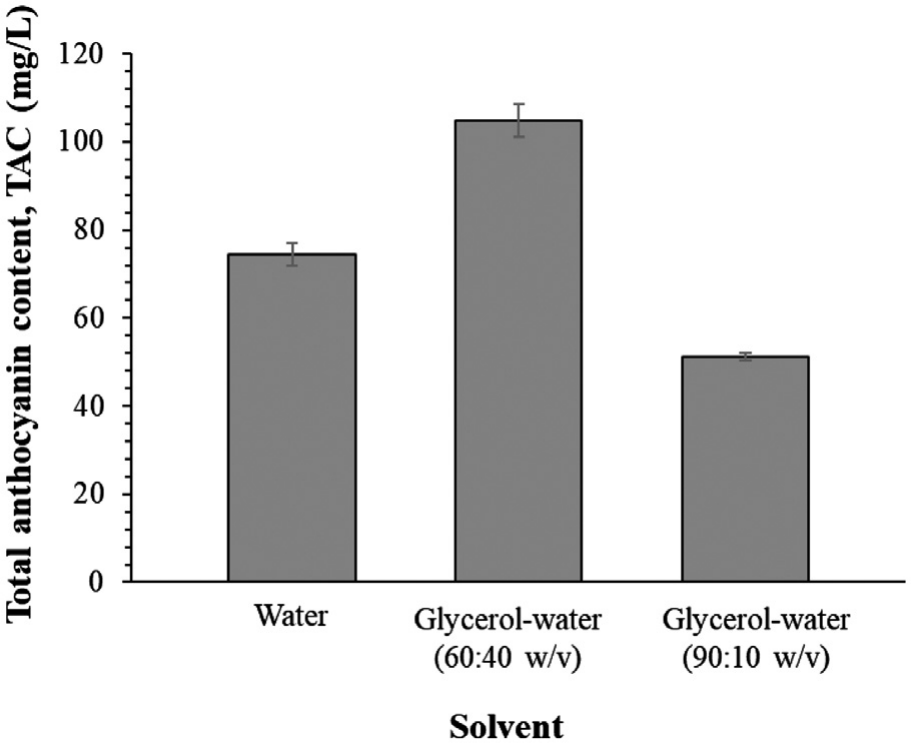
Extraction of anthocyanin using different types of green solvents. The extraction was performed at 40  °C for 15 min, using 10:1 of solvent to solid ratio. Data expressed as mg of anthocyanin per 1 L of extract. Data represented the mean value ± standard deviation (*n* = 3).

**Fig. 2:**
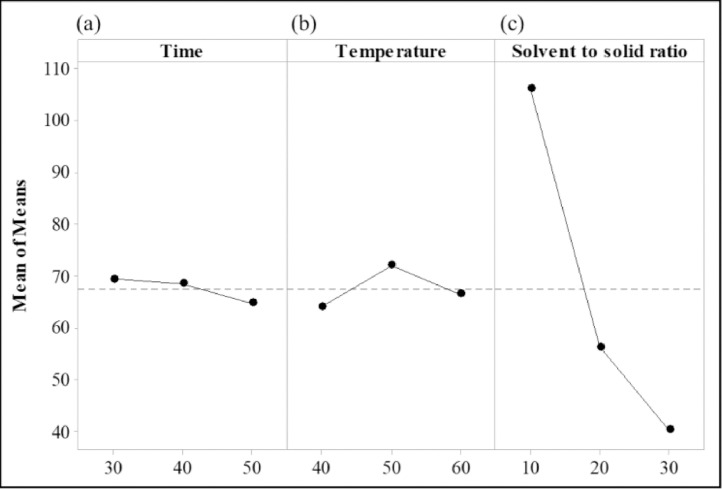
Main effects plot of 3 factors (a. Time, b. Temperature and c. Solvent to solid ratio) at three levels on respond, TAC.

**Fig. 3:**
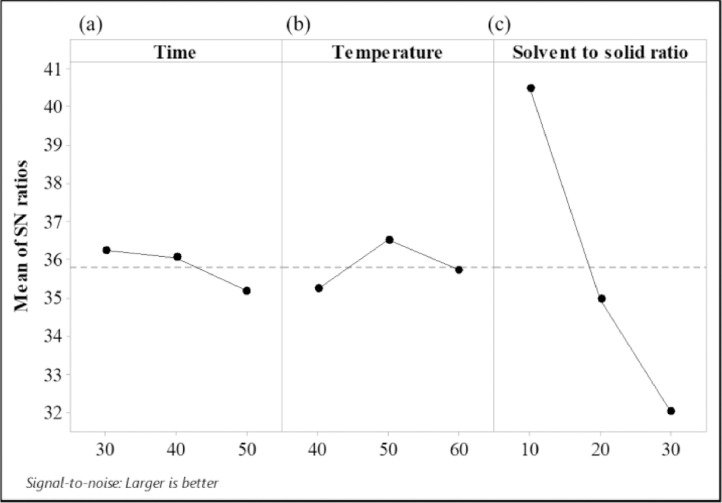
Effect of 3 factors (a. Time, b. Temperature, and c. Solvent to solid ratio) at three levels on S/N ratio.

Fig. 1 and Supplemental data 1 show the TAC in solvent screening process using UAE at 40 °C for 15 min, using 10:1 of solvent to solid ratio. Solvents used were water and glycerol-water (60:40 w/v and 90:10 w/v). Table 1 and Supplemental data 2 tabulate the TAC and S/N ratio data delivered from the Taguchi method with 27 experimental runs. The data in Table 2 describes the effects of factors estimated based on the means of TAC. Fig. 2 visualize the magnitudes of the main effects of factors. Data in Table 3 correspond to Fig. 3, which reports the interaction of 3 factors at 3 levels on the S/N ratio. The ANOVA and percentage of contribution for the 3 factors are displayed in Table 4. Table 5 and Supplemental data 3 illustrate the validation of predicted and experimental values (TAC and S/N ratio). Table 6 and Supplemental data 4 report the percentage of anthocyanin degradation in lighting conditions (fluorescent light and dark) and temperature (4 and 25 °C). Supplemental data 5 shows the lack of fit, model summary, coefficients, regression equation of S/N ratio, fits and diagnostics for unusual observations. The raw and processed dataset of solvent screening, Taguchi L27 experimental result, optimization validation, storage stability and ANOVA analysis are available in the Mendeley Data (https://data.mendeley.com/datasets/xp8jvh3t2x/1) as supplemental data.

**Table 1:** The L27 (3^3^) Taguchi method orthogonal matrix for UAE of anthocyanin from *C. ternatea*.

Experimental run	Time (min)	Temperature (°C)	Solvent to solid ratio (mL/g)	Total anthocyanin content (mg/L)	S/N ratio
1	30	40	10:1	100.82	40.07
2	30	40	10:1	101.52	40.13
3	30	40	10:1	106.72	40.57
4	30	50	20:1	61.08	35.72
5	30	50	20:1	64.35	36.17
6	30	50	20:1	62.65	35.94
7	30	60	30:1	40.80	32.21
8	30	60	30:1	42.43	32.55
9	30	60	30:1	43.78	32.82
10	40	40	20:1	59.03	35.42
11	40	40	20:1	53.60	34.58
12	40	40	20:1	52.89	34.47
13	40	50	30:1	42.55	32.58
14	40	50	30:1	41.15	32.29
15	40	50	10:1	49.74	33.93
16	40	60	10:1	105.03	40.43
17	40	60	10:1	107.60	40.64
18	40	60	10:1	104.97	40.42
19	50	40	30:1	35.48	31.00
20	50	40	30:1	31.56	29.98
21	50	40	30:1	35.54	31.01
22	50	50	10:1	107.89	40.66
23	50	50	10:1	108.71	40.73
24	50	50	10:1	110.23	40.85
25	50	60	20:1	48.22	33.66
26	50	60	20:1	50.67	34.10
27	50	60	20:1	54.65	34.75

**Table 2:** Response table for means of TAC.

	Factor		
Level	Time	Temperature	Solvent to solid ratio
1	69.35	64.13	105.94
2	68.51	72.04	56.35
3	64.77	66.46	40.33
Delta	4.58	7.91	65.61
Rank	3	2	1

**Table 3:** Response table for signal to noise ratio.

	Factor		
Level	Time	Temperature	Solvent to solid ratio
1	36.24	35.23	40.50
2	36.06	36.52	34.96
3	35.18	35.72	32.01
Delta	1.06	1.29	8.48
Rank	3	2	1

**Table 4:** ANOVA for UAE of anthocyanin.

Factor	Degree of freedom (DOF)	Sum of squares (SS)	Mean of squares (MS)	*F*-Value	*P*-Value	Percentage of contribution (%)
Time	2	5.762	2.881	13.08	0.00023	1.65
Temperature	2	7.662	3.831	17.40	0.00004	2.19
Solvent to solid ratio	2	331.721	165.861	753.19	0	94.90
Error	20	4.404	0.220			1.26
Total	26	349.549				100.00

**Table 5:** The predicted values and experimental data of TAC and S/N ratio prepared under the optimum condition. Experimental values presented as mean ± standard deviation (*n* = 3).

Response	Predicted value	Experimental value	Bias (%)
Total anthocyanin content (mg/L)	112.25	115.22 ± 4.20	2.58
S/N ratio	41.60	41.23 ± 0.32	0.91

**Table 6:** Effect of light and temperature on the percentage of anthocyanin degradation. Each of the values represented the mean ± standard deviation (*n* = 3).

	Percentage of anthocyanin degradation (%)			
	Lighting condition		Temperature	
Day	Fluorescent light	Dark	4 °C	Room temperature (25 °C)
0	0.00 ± 0.00	0.00 ± 0.00	0.00 ± 0.00	0.00 ± 0.00
1	3.35 ± 0.84	2.58 ± 0.58	2.39 ± 2.05	3.51 ± 2.62
3	8.14 ± 1.08	6.75 ± 1.32	3.85 ± 1.74	5.45 ± 2.94
6	12.50 ± 2.05	10.36 ± 1.65	8.38 ± 1.73	10.60 ± 2.59
7	15.61 ± 1.76	11.42 ± 1.78	10.56 ± 0.79	13.64 ± 2.33

## 2 Experimental Design, Materials and Methods

### 2.1 Materials

**Fig. 4:**
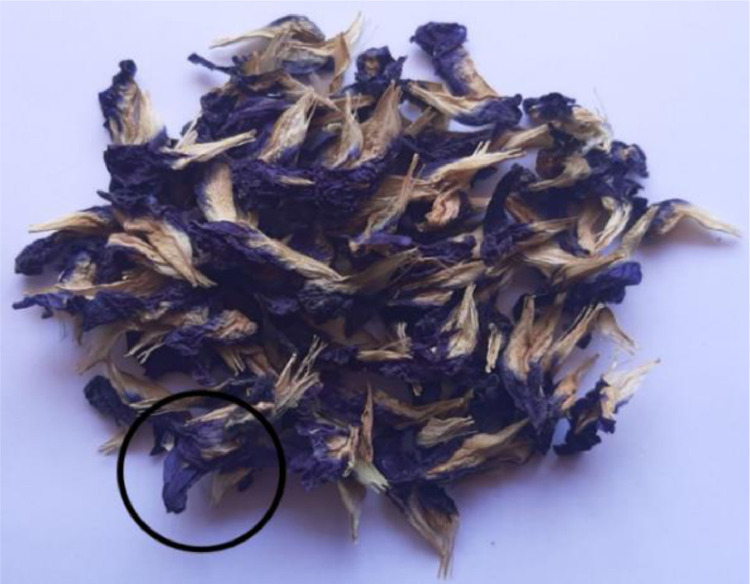
Blue color petals of *C. ternatea* (black circle) were utilized for extraction.

Fresh *C. ternatea* flowers were collected from a private residence in, Kedah, Malaysia. The fresh flowers were cleaned with tap water and dried under the sun. Only the blue color petals were selected (Fig. 4) and used for extraction purposes. The dried petals were ground and sieved to particle size below 710 μm. The sample was stored at 4 °C in a tightly sealed container until further use.

Glycerol with a purity of 99.5% (QReC, New Zealand) was used to prepare green solvent. Potassium chloride (Merck, Germany) was used to prepare pH 1.0 buffer solution. tri-Sodium citrate-2-hydrate (Riedel-de Haen, Germany) and citric acid (R&M Chemicals, United Kingdom) were used to prepare citrate buffer solution at pH 4.5.

### 2.2 UAE and solvent screening

Water and glycerol-water (60:40 w/v and 90:10 w/v) were used for the screening process. Extraction was conducted by adding 1 g of *C. ternatea* powder into three different solvents with a solvent to solid ratio of 10:1 and maintained at 40 °C for 15 min in an ultrasonic bath (Elmasonic, S80H) [1]. After the extraction time, the extract was vacuum filtered using filter paper, followed by centrifugation at 4000 rpm for 15 min. The supernatant was collected for further analysis. The solvent that produced the highest total anthocyanin content was used in the optimization and storage stability test. The extraction and solvent screening were performed in triplicate.

### 2.3 Determination of total anthocyanins content

The TAC in the *C. ternatea* extract was determined using the spectrophotometric pH differential method [2]. The *C. ternatea* extract was diluted separately with pH 1.0 and pH 4.5 buffer solutions. The absorbance value of equilibrated sample solution was measured at wavelength 550 and 700 nm using a UV-visible spectrophotometer (Agilent Technologies, Cary 60). The absorbance value of diluted sample (A) and TAC were calculated using Eqs. (1) and (2), respectively:

(1) $$A=\;\left(A_{550}-A_{700}\right)\text{pH}\;1.0-\;\left(A_{550}-A_{700}\right)\text{pH}\;4.5$$

(2) $$\text{Total}\;\text{anthocyanin}\;\text{content},\;\text{TAC}\;\left(\text{mg}/L\right)=\frac{A\;\times \;MW\;\times \;DF\;\times \;1000}{\varepsilon \;\times \;l}\;$$

Where MW is the molecular weight of the anthocyanins reference pigment (cyanidin-3-glucoside, MW = 449.2 g/mol), DF is the dilution factor, ε is the molar absorptivity of the reference pigment (cyanidin-3-glucoside, ε = 26 900 L mol^−1^ cm^−1^), and l is the cuvette width (1 cm). The measurement of TAC was done in triplicate.

### 2.4 Experimental design

Taguchi method was adopted in this work to optimize the extraction condition. The three factors (extraction time, extraction temperature, and solvent to solid ratio), with three levels setting, were considered as independent variables and summarised in Table 7. An L27 (3^3^) orthogonal matrix showed 27 experimental runs derived from 3 factors (columns) with 3 levels (rows). The TAC (mg/L) was evaluated at the end of each experiment. The function higher the better was selected as maximizing the TAC was the main objective in this experiment. The function was represented as Signal-to-Noise (S/N) ratio and determined according to Eq. (3):

(3) $$S/N=-10\log\left(\frac{1}{n}\sum_{i=1}^{n}\frac{1}{y_{i}^{2}}\right)$$

Where n is the number of repetitions for an experimental combination, and y*_i_* is a performance value of the *i*th experiment. The extracts were prepared in triplicate.

**Table 7:** Factors and levels of Taguchi L27 orthogonal design.

	Level		
	1	2	3
Extraction time (min)	30	40	50
Extraction temperature (°C)	40	50	60
Solvent to solid ratio	10:1	20:1	30:1

The importance of factors on TAC was based on the delta value, and the highest S/N ratio for each factor at the particular level was selected as the optimum extraction parameter [3].

### 2.5 Validation of experimental and predicted data

The experimental and predicted value of TAC and S/N ratio under optimum conditions were validated based on the percentage of bias [4] as stated in Eq. (4). The experiment value was done in triplicate.

(4) $$\text{Percentage}\;\text{of}\;\text{bias}\;\left(\%\right)=\frac{\text{Predicted}\;\text{value}-\text{Experimental}\;\text{value}\;}{\text{Experimental}\;\text{value}}\times \;100\%\;$$

### 2.6 Anthocyanin's storage stability test

The effect of light on the stability of anthocyanin was studied in two conditions at room temperature. For dark condition, the *C. ternatea* extracts were stored in a tightly sealed glass bottle and were fully covered with aluminum foil. For light condition, the glass bottle was directly exposed to fluorescent light (Philips Lifemax, TLD 36 W/765, cool daylight). The effect of temperature was investigated at 4 °C and room temperature (25 °C). The extracts were stored in tightly sealed glass bottles wrapped with aluminum foils. The extracts were prepared in triplicate. The absorbance of all extract was measured at wavelength 550 nm using a UV-visible spectrophotometer (Agilent Technologies, Cary 60) on day 0, 1, 3, 6, and 7 of storage. The absorbance was used to determine the percentage of anthocyanin degradation using Eq. (5), according to Askar et al. (2015) [5]:

(5) $$\text{Anthocyanin}\;\text{degradation}\;\left(\%\right)\;=\;\frac{A_{\text{initial}}-A_{\text{final}}}{A_{\text{initial}}}\times \;100\%\;$$

Where A_initial_ is the initial absorbance of the sample solution, and A_final_ is the absorbance at a specific time interval at different storage conditions.

### 2.7 Statistical analysis

All experiments were carried out in triplicates. The results of TAC and the percentage of anthocyanin degradation were expressed as the mean ± standard deviation. Taguchi experimental design was performed by Minitab 18 statistical software (Minitab, State College, PA, USA). Analysis of data was conducted using ANOVA to evaluate the significance of the independent variables and their interactions. The difference was considered statistically significant when the *p*-value ≤ 0.05.

## CRediT authorship contribution statement

**Meng Hong Shu:** Writing – original draft, Writing – review & editing. **Kanagesvari K. Annamalai:** Methodology, Conceptualization, Formal analysis, Validation, Data curation, Investigation. **Farhana Nazira Idris:** Supervision, Resources. **Azlina Harun Kamaruddin:** Resources. **Masrina Mohd Nadzir:** Supervision, Writing – review & editing, Resources, Funding acquisition.

## Declaration of Competing Interest

The authors declare that they have no known competing financial interests or personal relationships which have, or could be perceived to have, influenced the work reported in this article.
